# Optimization of fungicidal and acaricidal metabolite production by endophytic fungus *Aspergillus* sp. SPH2

**DOI:** 10.1186/s40643-024-00745-9

**Published:** 2024-03-05

**Authors:** Nicolas Reyes Castillo, Carmen E. Díaz, M. Fe Andres, Juan Imperial, Félix Valcárcel, Ana Azucena González Coloma

**Affiliations:** 1 Consejo Superior de Investigaciones Científicas (CSIC), Instituto de Ciencias Agrarias (ICA), Calle de Serrano 115B, 28006 Madrid, Spain; 2 Instituto de Productos Naturales y Agrobiología (IPNA) - CSIC, Avda. Astrofísico F. Sánchez, 3, Tenerife, 38206 La Laguna, Spain; 3 Centro de Biotecnología y Genómica de Plantas, Universidad Politécnica de Madrid (UPM), Instituto Nacional de Investigación y Tecnología Agraria y Alimentaria (INIA/CSIC), Campus de Montegancedo UPM, Pozuelo de Alarcón, 28223 Madrid, Spain; 4 Grupo de Parasitología Animal, Departamento de Reproducción Animal, INIA-CSIC, Carretera de La Coruña, Km 5,9, 28040 Madrid, Spain; 5 Grupo de Trabajo ESGARIBER, Sociedad Española de Parasitología,, Plaza de Ramón y Cajal s/n, Ciudad Universitaria, 28040 Madrid, Spain

**Keywords:** Endophyte, *Aspergillus*, Antifungal, Ixodicidal, Fermentation, Mellein

## Abstract

The endophytic fungus *Aspergillus* sp. SPH2 was isolated from the stems of the endemic plant *Bethencourtia palmensis* and its extracts were found to have strong fungicidal effects against *Botrytis cinerea* and ixodicidal effects against *Hyalomma lusitanicum* at different fermentation times. In this study, the fungus was grown using three different culture media and two methodologies, Microparticulate Enhancement Cultivation (MPEC) and Semi-Solid-State Fermentation (Semi-SSF), to increase the production of secondary metabolites during submerged fermentation. The addition of an inert support to the culture medium (Semi-SSF) resulted in a significant increase in the extract production. However, when talcum powder was added to different culture media, unexpected results were observed, with a decrease in the production of the biocompounds of interest. Metabolomic analyses showed that the production of aspergillic, neoaspergillic, and neohydroxyaspergillic acids peaked in the first few days of fermentation, with notable differences observed among the methodologies and culture media. Mellein production was particularly affected by the addition of an inert support to the culture medium. These results highlight the importance of surface properties and morphology of spores and mycelia during fermentation by this fungal species.

**Graphical Abstract:**

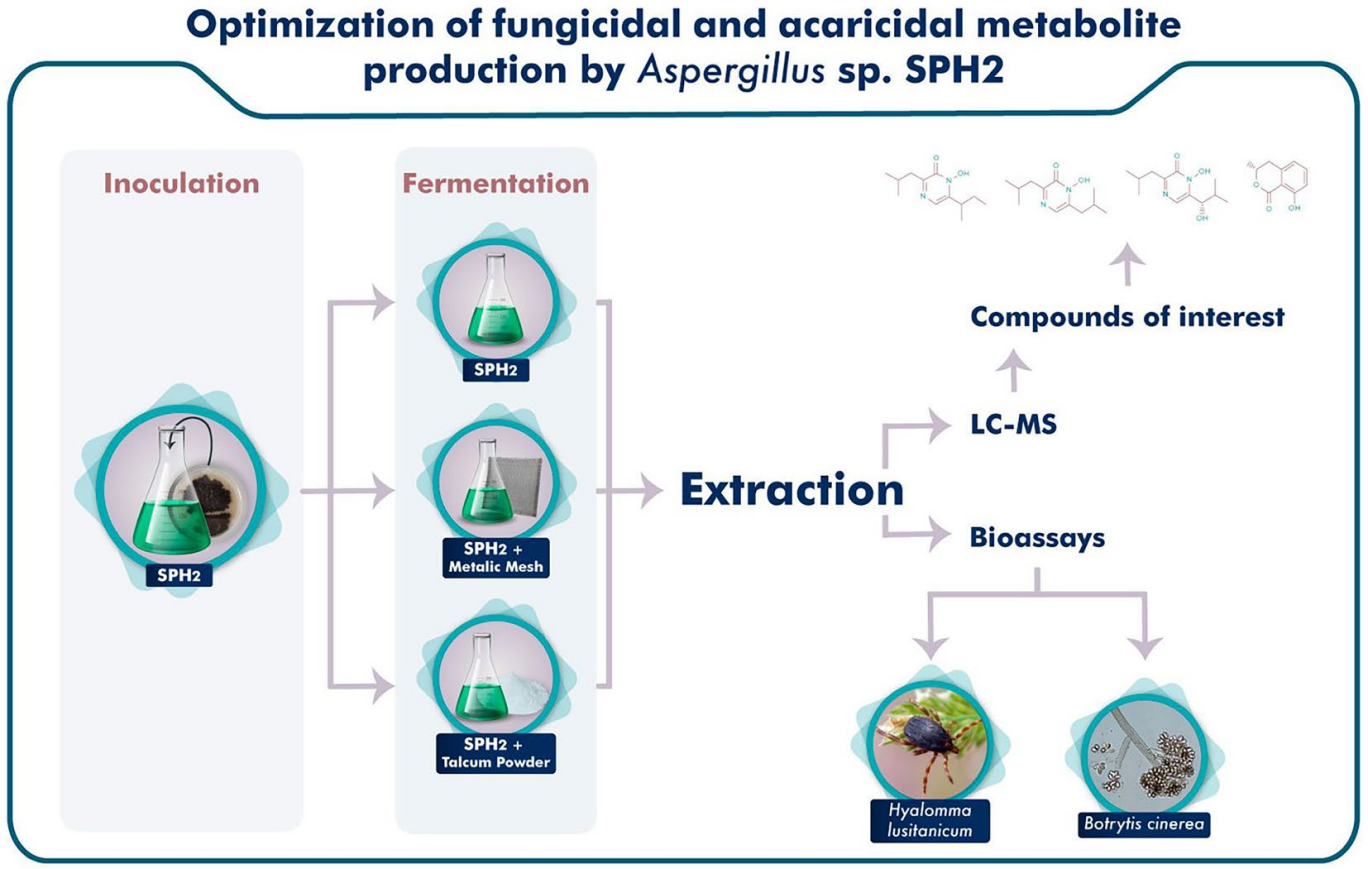

## Introduction

Currently, agriculture is facing the global challenge of being productive, efficient, sustainable, and environmentally friendly (Baker et al. 2020). Food production is affected by plant diseases and insect pests, causing millions of losses and jeopardizing food security (Kumar and Singh 2015). To date, plant protection has depended on synthetic pesticides, resulting in several direct negative effects on farmer and consumer health, soil erosion, water quality, and a number of associated problems such as the emergence of pest resistance. In this context, there has been an increase in the research, development, and application of bio-pesticides because of their efficacy, harmlessness to the population and environment, and target-specific properties (Damalas and Koutroubas 2018; Samada and Tambunan 2020). Additionally, there has been an increase in the demand for food safety, product quality, and stricter pesticide regulations, making the search for new bio-pesticides a priority (Damalas and Koutroubas 2018).

Microorganisms, including endophytes, which live within the internal tissues of plants and their secondary metabolites, are a source of biopesticides (Tawfike et al. 2017). *Bethencourtia*, a genus found exclusively on the Canary Islands, includes three distinct species: *B. hermosae* (Pit), *B. palmensis* (Nees) Choisy, and *B. rupicola* (B. Nord) B. Nord (Nordenstam 2006). Endophytes from this plant specie have been reported to exhibit insect antifeedant activities, that’s due to *B. palmensis* contains silphinene sesquiterpenes with potent insect antifeedant effects (Portero et al. 2012). Based on these results, this plant was selected for the isolation of endophytic fungi to identify the secondary metabolites with biopesticidal properties. In this context, the endophyte *Aspergillus sp.* SPH2 has been isolated from *B. palmensis* (Morales-Sánchez et al. 2021)*.* The production of secondary compounds with biopesticidal potential is based on the recognition of endophytic fungi as prolific producers of compounds effective against pathogens and herbivores, also Some studies have reported production of compounds with low molecular weight, that have antiviral, antifungal, and antibacterial properties and affect insect growth regulators (Latz et al. 2018; Berestetskiy and Hu 2021), these compounds comprises a wide range of chemical classes, including alkaloids, steroids, terpenoids, peptides, polyketones, flavonoids, quinols, phenols, chlorinated compounds, and volatile organic compounds (VOCs) (Baron and Rigobelo 2022). Notably, the endophytic fungal isolate *Aspergillus sp*. Recently, SPH2 has been shown to produce mellein and neoaspergillic acid at different stages of fermentation (Morales-Sánchez et al. 2021). Mellein is a subgroup of 3,4-dihydroisocoumarins; usually, secondary metabolites belonging to the polyketide group; with antimicrobial and phytotoxic activities (Reveglia et al. 2020) and efficacy against the disease vector *Hyalomma lusitanicum* ticks (Morales-Sánchez et al. 2021). Neoaspergillic acid is a siderophore (MacDonald 1970) with fungicidal activity against *Botrytis cinerea* (Morales-Sánchez et al. 2021). Therefore, optimization of the fermentation conditions to produce these metabolites could increase the potential for the development of bio-based pesticides.

Fungal fermentation and its optimization play a crucial role in the production of compounds of interest, both in terms of the controlled environment and method used. Submerged fermentation (SmF) and solid fermentation (SSF) are the most widely used methods and offer unique advantages and disadvantages (Suriya et al. 2016; Sharma et al. 2017; Ouedraogo and Tsang 2021); SSF has the potential to reduce costs and energy consumption by utilizing agro-industrial waste (Sala et al. 2021), depending on the case, produces low waste water production and high product stability (Cruz et al. 2015), but traditional SSF systems cannot provide sufficient mass and heat transfer which is crucial in these processes (Yang 2007). On the other hand, SmF has benefits such as higher productivity and yields (Ramos and Malcata 2011; Moresi and Parente 2014), also in controlling and measuring of parameters is simpler than SSF (Reihani and Khosravi-Darani 2019). The primary challenge in submerged fungal fermentation is the development of intricate mycelial clumps or pellets. The presence of these pellets results in an increased viscosity of the media, which in turn impedes the effective transfer of oxygen and nutrient resources in the liquid phase.. (Iram et al. 2022). Other factors that play a significant role in the choice of fermentation method, in addition to physical factors, include the availability of the substrate to the fungus, morphology of the fungus, impact of the biofilm, and effects of the mass (Pandey 1992). Semi-solid-state fermentation (Semi-SSF) is a hybrid method that utilizes both an inert solid support and a smaller volume of culture medium. This technique is considered sustainable and cost-effective (Borah et al. 2023).

Considering that the morphology of the fungus in submerged media affects its productivity, semi-solid-state fermentation (Semi-SSF), in which the fungus grows in a biofilm, allows the production of secondary metabolites in high quantities (Barrios-González 2012; Francis et al. 2020). This method involves the use of techniques that impede the formation of fungal agglomerates, resulting in smaller, less dense pellets or even dispersion of the mycelium throughout the growth medium (Kowalska et al. 2018), leading to more efficient substrate consumption, much greater oxygen transfer, and increased productivity of the fungus. The most widely used technique is microparticle-enhanced cultivation (MPEC) using talcum powder or aluminum oxide (Walisko et al. 2012). The media may be rich in nutrients and optimally aerated, but the cells inside the sphere are stressed by limitations in the exchange of oxygen and nutrients (Gibbs et al. 2000), because effective growth occurs exclusively on the surface of the mycelium aggregate.

In this study, the application of talcum powder as an MPEC method as well as the addition of a metallic mesh to the culture medium as a Semi-SSF method have been evaluated to observe changes in the production of mellein, aspergillic, neohydroxyaspergillic, and neoaspergillic acids by the endophyte fungus *Aspergillus sp.* SPH2.

## Results

### Appearance and coloring of SPH2 mycelium

The modifications carried out in the different fermentations resulted in changes in the coloration and aggregation patterns of the fungal pellets as well as their size and general transformation over time (Table 1).

**Table 1:** Changes in SPH2 pellet formation and coloring during fermentation

Culture media	Modification	Characteristics
PDB	None	Homogeneous pellets, reddish color
	Talcum Powder	Pellet agglomeration, red-brown color
	Metallic Mesh	Mycelium on mesh, light brown color
CZD	None	Homogeneous pellets, no change in color
	Talcum Powder	Pellet agglomeration, light red color
	Metallic Mesh	Mycelium clumps on mesh, reddish color
CZDM	None	Non-homogeneous pellet agglomeration, yellowish red
	Talcum Powder	Variable-sized pellets, light red color
	Metallic Mesh	Pellet clustering on mesh, strong orange color

### pH measurement

**Fig. 1:**
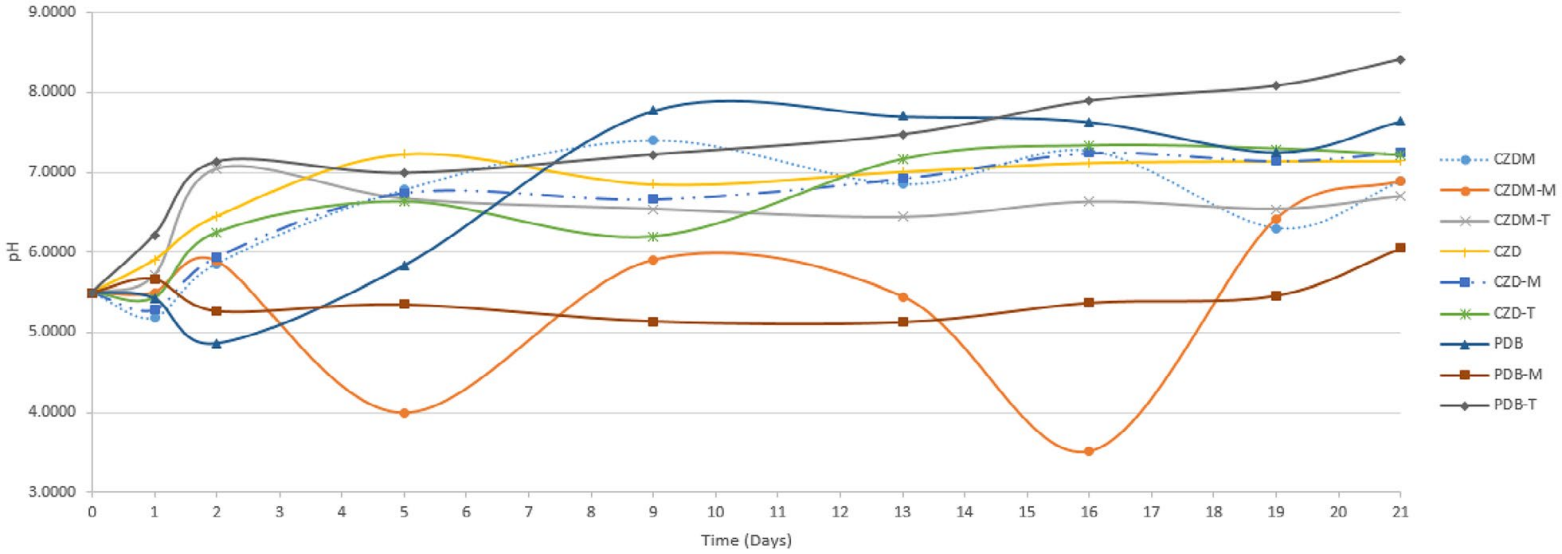
Time course of SPH2 pH

The time course of fermentation of the SPH2 fungus showed a minimum pH of 4 and 3.52, on days 5 and 16 in the CZDM culture medium, respectively. With the addition of an inert support (metallic mesh), the maximum pH of 8.41. was observed on day 21 of fermentation in PDB and talcum powder was added (Fig. 1).

### Mycelium yield

**Fig. 2:**
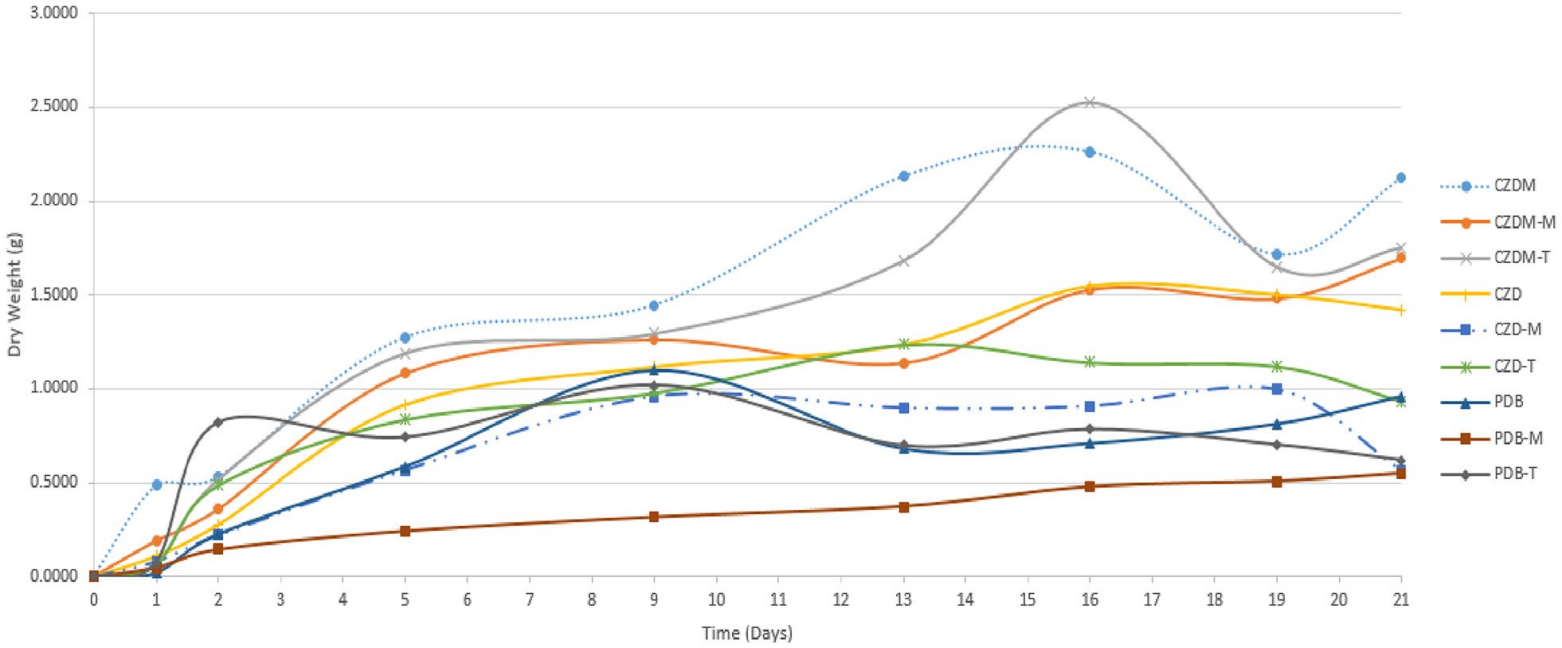
Time course of SPH2 mycelial yield

The lowest mycelium dry weight values were found for PDB-M, whereas CZDM, CZDM-T, and CZDM-M showed significant increases (2.1234 g, 1.7529 g, and 1.6968 g, respectively). The general trend observed for the dry weight yield of the remaining fermentations was similar over time, with PDB-T yielding the lowest yield (0.6195 g) and CZD the highest (1.4199 g) (Fig. 2).

### Extract yield

**Fig. 3:**
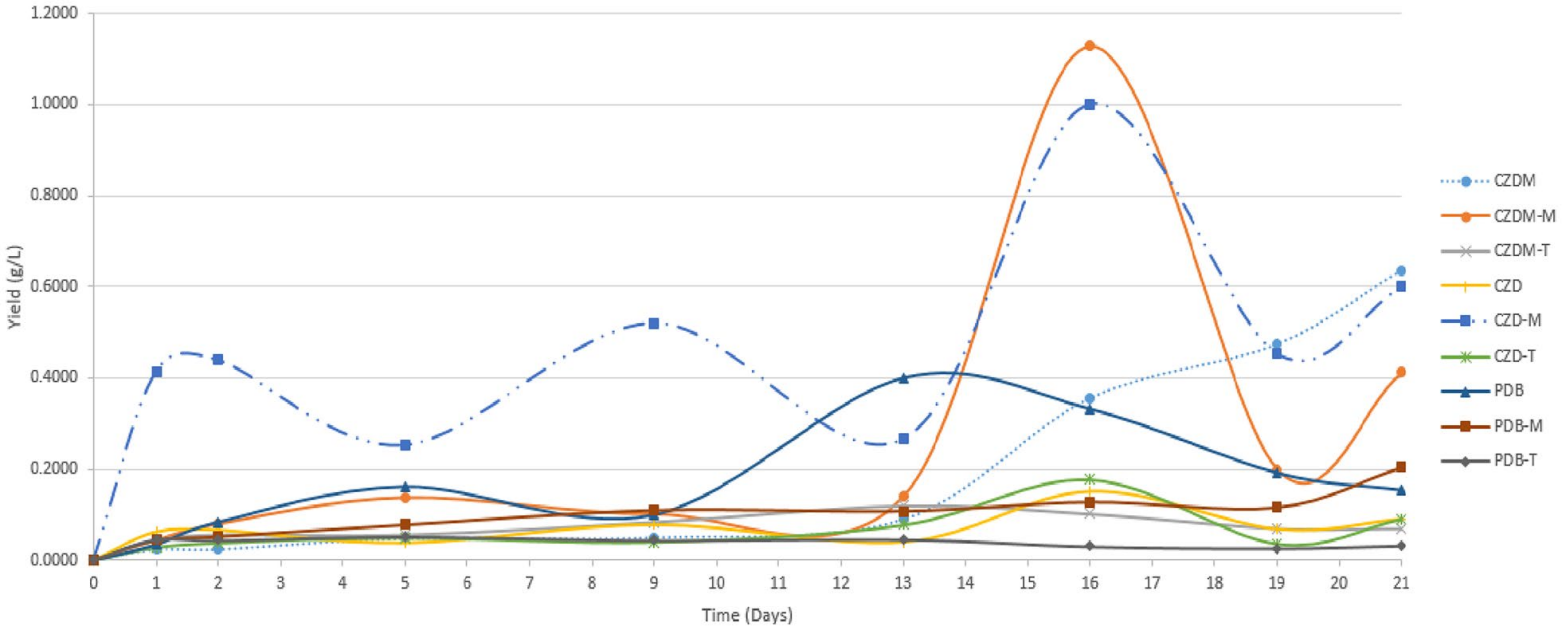
Time course of SPH2 extract yield

The CZD-M medium showed superior performance for all samples in the first 9 days of measurement, while CZDM-M gave the highest yield value on day 16 (1.1280 g/L); however, on day 21 of fermentation, the yield dropped to 0.4120 g/L, while CZDM and CZDM-M yielded 0.6360 g/L and 0.6000 g/L, respectively. The performance of PDB medium in stationary fermentation was prolonged over time, although the yield was not the highest. The rest of the fermentations showed similar performance, not exceeding 0.2040 g/L (maximum value achieved at 21 days for PDB-M) throughout the incubation period (Fig. 3).

### Secondary metabolite analysis

**Fig. 4:**
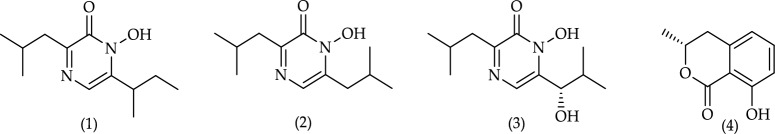
Compounds of interest: (**1**) Aspergillic acid, (**2**) neoaspergillic acid, (**3**) neohydroxyaspergillic acid, and (**4**) mellein

Fungi are the most important source of melleins, with (*R*)-(-)-mellein (**1**) being the most common among these groups (Reveglia et al. 2020). Mellein showed ixodicidal activity, and aspergillic (**2**), neoaspergillic (**3**), and neohydroxyaspergillic (**4**) acids showed antifungal activity (Morales-Sánchez et al. 2021). Therefore, these compounds were selected to monitor the fermentation optimization process. Figure 4 shows the chemical structures of these compounds.

**Fig. 5:**
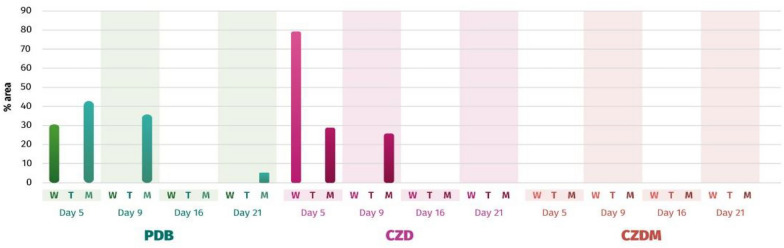
Quantification of aspergillic acid (**1**) over time (days 5, 9, 16, and 21) in different culture media (W = without any modification, T = with talcum powder, and M = with metallic mesh)

The selected secondary metabolites were analyzed in various culture media using GC–MS at specific time points during the fungal growth curve. The chosen time points included the onset of the exponential phase (day 5), the transition from the exponential phase to the stationary phase (day 9), the transition from the stationary phase to the senescent phase (day 16), and the fermentation endpoint (day 21). The production of aspergillic acid (**1**) showed two peaks on days 5 and 9 in the CZD and PDB (Fig. 5). Incubation with CZD increased production by 1 to 30% on day 5 compared to PDB in the absence of metallic mesh. It's also worth noting that 1 was absent in the CZDM medium. Furthermore, consistent production of this compound appeared to be concentrated within the initial 9-day period.

**Fig. 6:**
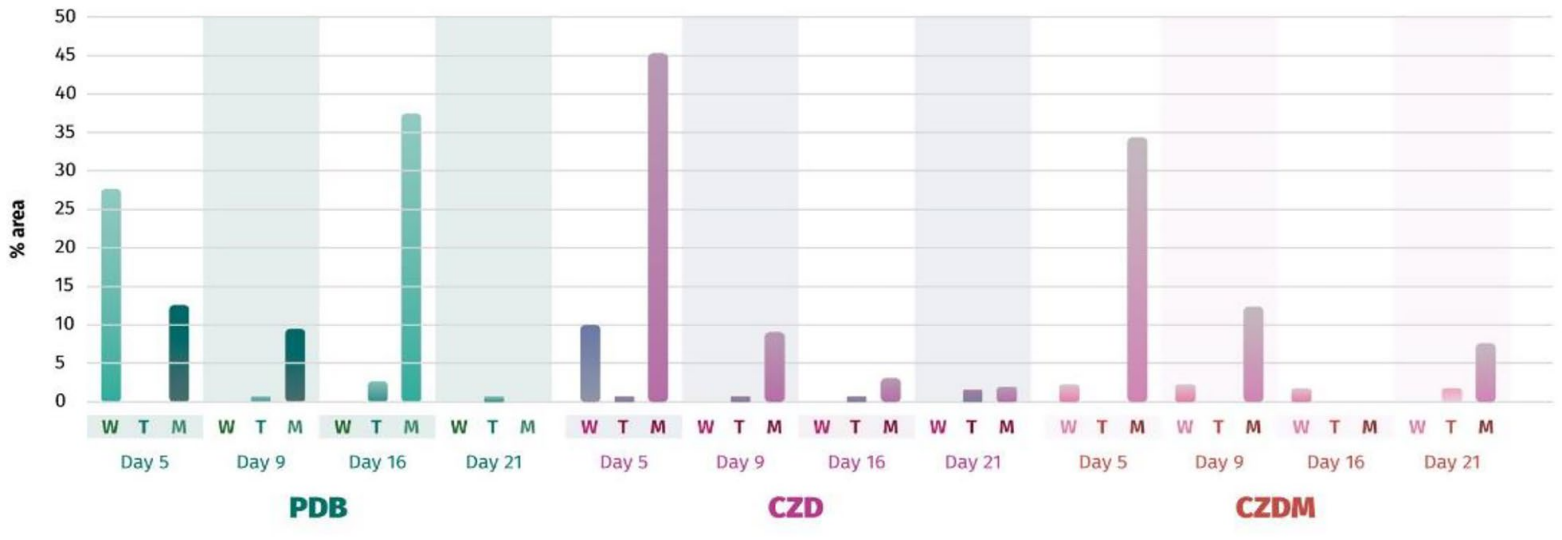
Quantification of neoaspergillic acid (**2**) over time (days 5, 9, 16 and 21) in different culture media (W = without any modification, T = with talcum powder, and M = with metallic mesh)

Figure 6 shows the production of neoaspergillic acid (**2**), with the maximum value on day 5 instead of day 8, as previously reported (Morales-Sánchez et al. 2021). The addition of talcum to the PDB culture medium reduced production by 2-to 10 times. Similarly, the addition of talcum powder to CZD medium resulted in poor yields. In PDB medium, the synthesis of **2** increased by 20% in the presence of metallic mesh on day 16 compared to a previous study (Morales-Sánchez et al. 2021), and also increased on day 5 in CZD and CZDM media.

**Fig. 7:**
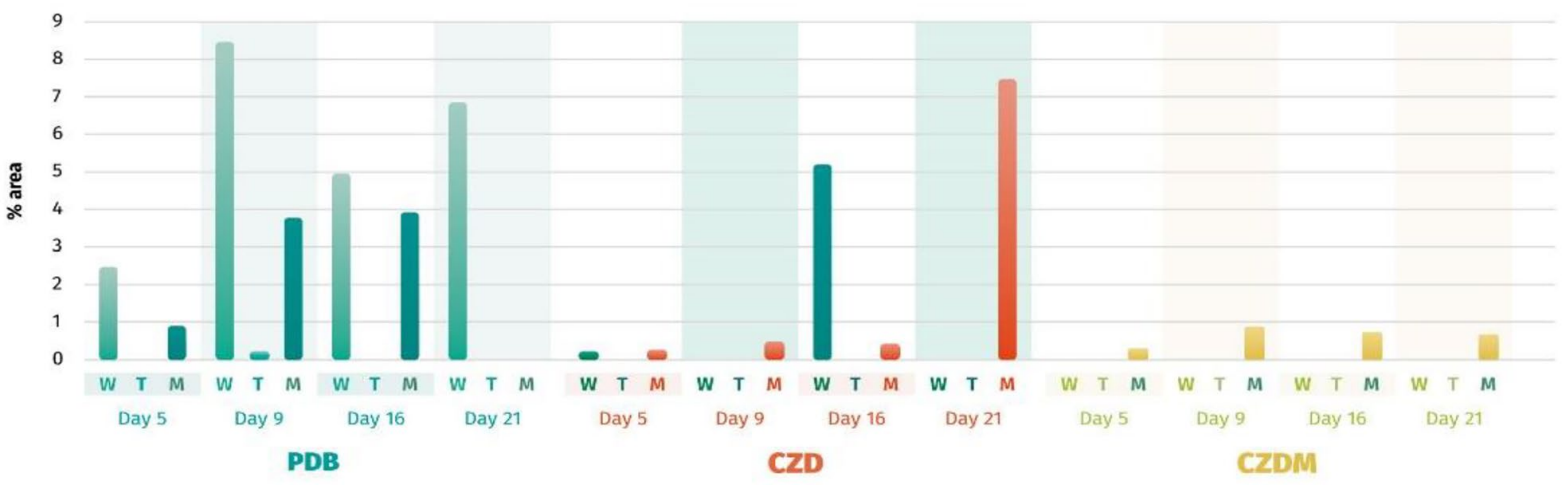
GC–MS quantification of neohydroxyaspergillic acid (**3**) over time (days 5, 9, 16 and 21) in different culture media (W = without any modification, T = with talcum powder and M = with metallic mesh)

Figure 7 shows the production of neohydroxyaspergillic (**3**), which was notably enhanced in the PDB culture medium. Without any modifications to the medium, a production yield of approximately 5–8% was achieved between days 9 and 21. To match this yield, CZD culture medium required the incorporation of a metallic mesh, which was observed on day 21 of fermentation. In the CZDM culture medium, the addition of a metallic mesh triggered the production of **3** in trace amounts.

**Fig. 8:**
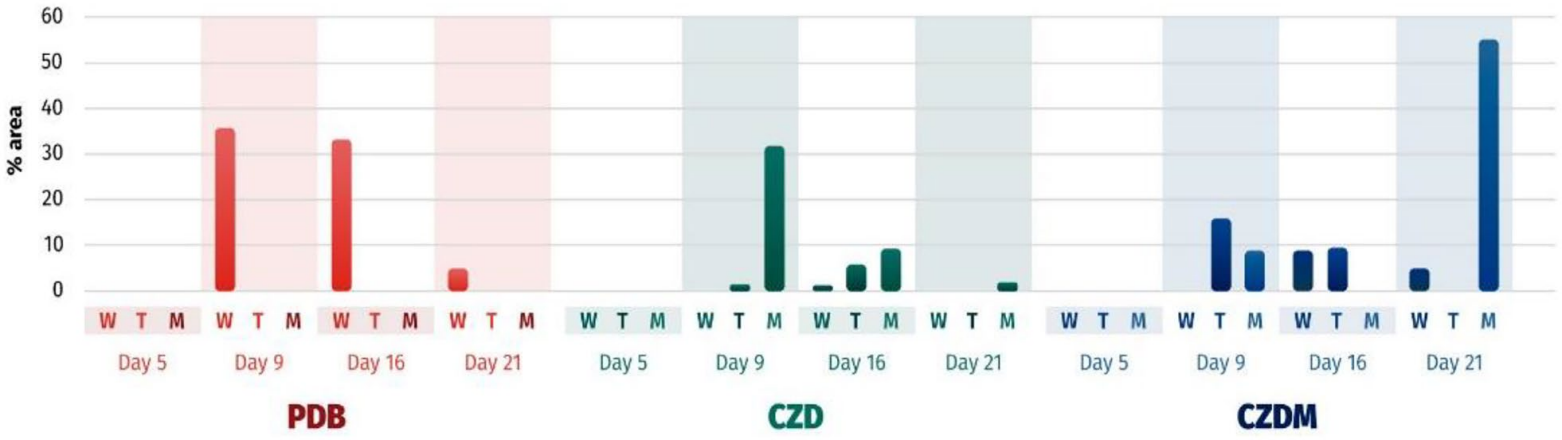
GC–MS quantification of mellein (**4**) over time (days 5, 9, 16, and 21) in different culture media (W = without any modification, T = with talcum powder, and M = with metallic mesh)

The production of mellein (**4**) (Fig. 8) ranged between 36–2% between days 9–21; while a range of 4–18% between days 7–13 was found previously (Morales-Sánchez et al. 2021), indicating that the production of this targeted compound starts between days 5 and 9 in fermentation with PDB. Observations from Fig. 8 indicated that the production of compound **4** was altered by the introduction of inert supports. The effects of talcum and metallic mesh were different. A yield of approximately 35% was recorded between days 9 and 16 in the PDB culture medium without any support. In contrast, when metallic mesh was used, similar yields were observed on day 9 with CZD and on day 21 with CZDM (60% yield).

### Cluster analysis for secondary metabolites: extract selection

The extracts were grouped into discrete categories based on their relative percentage areas as determined by GC–MS for compounds **1**,** 2**,** 3**, and **4**. The objective of this classification was to identify homogeneous clusters for future bioassay-based studies.

**Fig. 9:**
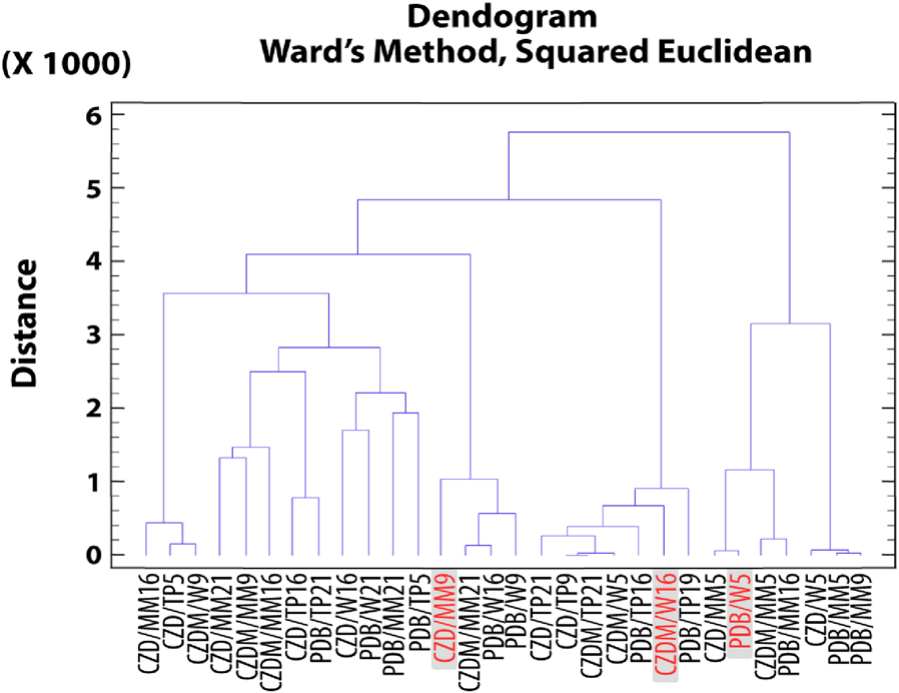
Dendrograms of compounds 1, 2, and 3. Highlighted are the chosen extracts, CZD/MM9, CZDM/W16 and PDB/W5

Figure 9 shows a dendrogram constructed based on the proportions of antifungals compounds **1**, **2**, and **3**. The clustering algorithm considers the relative percentage area of each compound, thereby defining the selection parameters for prospective application as antifungal agents. Following this clustering, three representative branches from each cluster were chosen for comprehensive analysis.

Based on this analysis, it was possible to identify compounds that exhibited higher yields in production, considering the sum of compounds **1**, **2**, and **3** in relation to the extract production. This determination was made according to Eq. (1), as outlined below:

1 $$\left(\sum_{Compound\, 1}^{Compound \,3}\mathrm{\% }\,Area\right)*Extract\, production\, of\, each\, fermentation$$

**Fig. 10:**
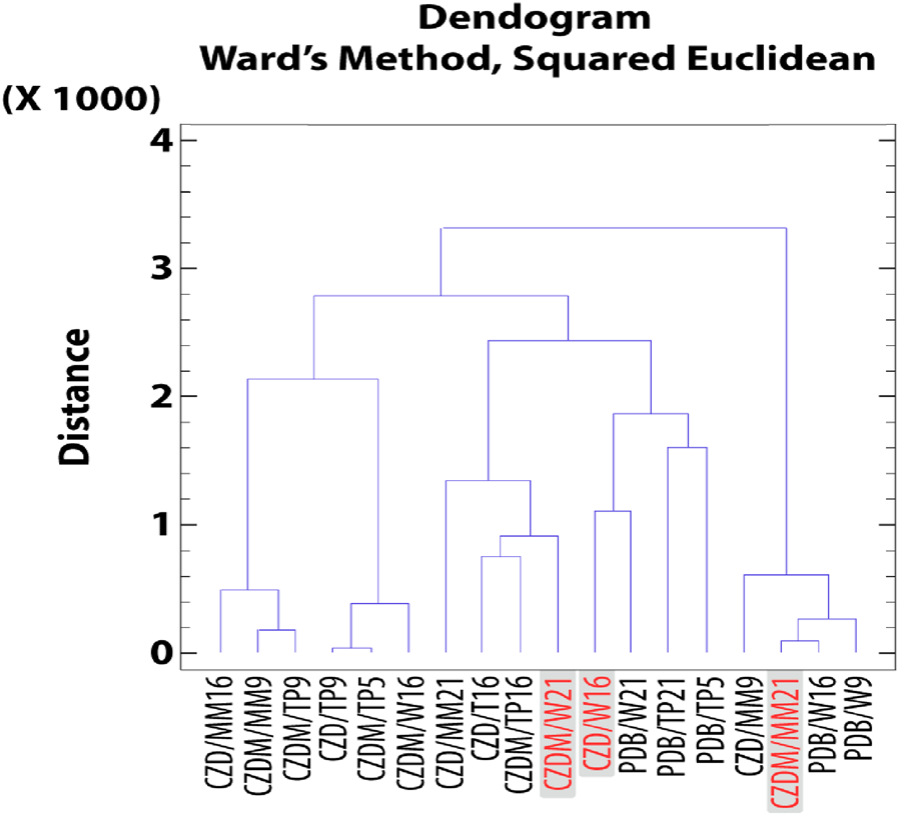
Dendrogram based on compound **4**. Highlighted are the chosen extracts, CZDM/W21, CZD/W16 and CZDM/MM21

Considering these results, the possible antifungal candidates are PDB/W5, CZDM/W16, and CZD/MM9, which are highlighted in red in Fig. 9. Subsequently, the same clustering process was performed for compound **4** (Fig. 10).

Based on the analysis conducted using the formula outlined below (2), the extracts to be evaluated were correctly established.

2 $$\left(\sum \mathrm{\% }area\, compound\, 4\right)*Extract\, production\, of\, each\, fermentation$$

From this indicator, as depicted in the dendrogram in Fig. 10, extracts delineated in red, such as CZDM/MM21, CZDM/W21, and CZD/W21, emerged as prospective candidates exhibiting ixodicidal activity attributable to compound **4**.

### Bioassays

Compounds **1**, **2**, and **3** were tested against *Botrytis cinerea* Pers. (1794), whereas the **4**-based cluster were evaluated against *Hyalomma lusitanicum* Koch. (1844).

### Spore germination inhibition bioassay

**Fig. 11:**
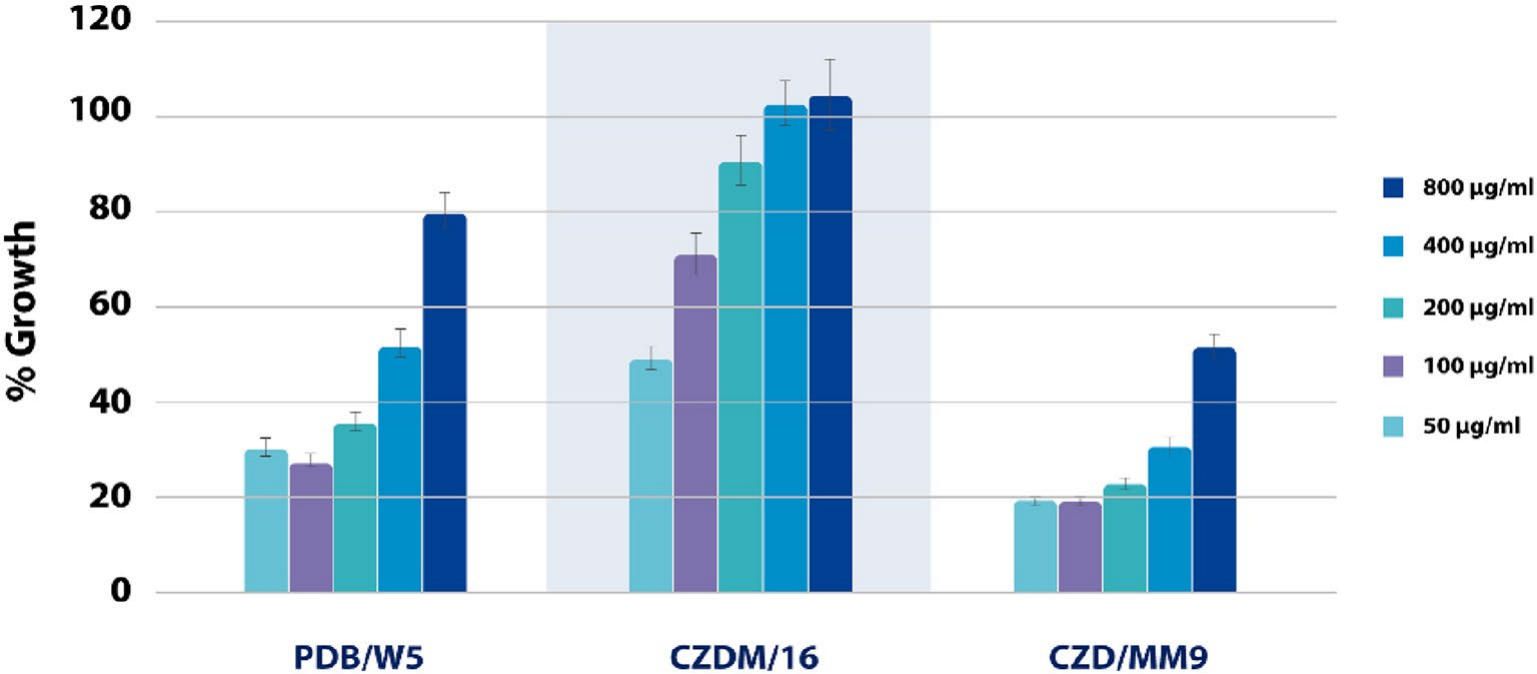
Growth rates in *the Botrytis cinerea* germination inhibition bioassay

Figure 11 shows the percentage inhibition of spore germination at different doses of the selected extracts. The CZD/MM9 extract was the most active (inhibition values of 80.98, 81.17, 77.46 and 69.71 at 800, 400, 200 and 100 µg/mL), followed by PDB/W5 (inhibition values of 69.81, 72.56, and 64.48 at 800, 400, and 200 µg/mL) with EC_50_ values of 0.0027 and 0.0150 µg/mL respectively (Table 2).

**Table 2:** Efficient doses of the antifungal SPH2 extract against *Botrytis cinerea s*pore germination

Extract	EC_50_ (μg/mg)	95% Confidence Limits
CZD/MM9	27.17	14.84–49.86
PDB/W5	149.75	116.49–192.38
CZDM/W16	> 40	> 40
*Aspergillus sp.* SPH2 (Morales-Sánchez et al. 2021)	22.00	19.00–26.00

### Ixodicidal bioassay

Table 3 shows the ixodicidal effects of **4**-based selected extracts (CZDM/MM21, CZDM/W21, CZD/W16). The most active extracts were CZDM/MM21, followed by CZDM/W21, with calculated lethal doses (LD_50_) of 5 and 28 μg/mg, respectively.

**Table 3:** *Hyalomma lusitanicum* larval mortality of selected SPH2 extracts

Extract	Mortality Rate (%)	LD_50_ (μg/mg)	95% confidence Limits
CZDM/MM21	100.00	5.00	4.4—5.66
CZDM/W21	100.00	28.08	25.16—31.26
CZD/W16	12.10	> 40	> 40
*Aspergillus sp.* SPH2 (Morales-Sánchez et al. 2021)	100.00	7.18	6.67 – 7.78
Mellein (4) (Morales-Sánchez et al. 2021)	100.00	0.48	0.44 – 0.51

## Discussion

In the present study, different fermentation parameters were tested for *Aspergillus* sp. SPH2 (isolated from *B. palmensis*) has been shown to produce bioactive metabolites (Morales-Sánchez et al. 2021). The fungal media PDB, CZD, and CZDM were modified using two physical methods (microparticle enhancement culture with talcum powder, MPEC, and the addition of a metallic mesh to the culture medium, Semi-SSF), resulting in changes in both pH and dry mycelium weight.

The results with talcum powder (MPEC) showed the formation of pellets of regular and homogeneous size in the first growth stage and a more pronounced agglomeration in the second stage (around day 13 of fermentation), which could be an indication of changes in production, as studies suggest that at a smaller particle size, the yield increases considerably (Singh 2018). The generation of different oxygen and mass transfer profiles that occur with the addition of talcum powder generally leads to the production of higher amounts of secondary metabolites (Gonciarz and Bizukojc 2014). In this study, secondary metabolites were produced, but not in the expected amounts. Therefore, microparticle enhancement culture (MPEC) should guarantee a pellet size that is sufficiently small to avoid agglomeration over time to ensure the correct transfer of oxygen and mass in the surface layer of the fungus. These results differ significantly from those reported (Niu et al. 2020) showing promising results with the use of 45 μm talcum powder for the production of echinocandin B. It also shows that the particle size may be responsible for most of the increase in productivity, since there was also an increase in the production of β-mannanase using 10 μm talcum powder (Germec et al. 2017). Additionally, smaller particles sizes interacted better with fungal growth, generating a positive response in *Chaetomium globosum* DX-THS3 (Du et al. 2020).

The addition of a metallic mesh (Semi-SSF) results in the formation of pellet agglomerations during both stages of growth. In the first stage, irregularly sized and shaped pellets aggregated around the metallic mesh. In the second stage, all pellets agglomerated, resulting in the formation of round, hairy pellets. These observations are consistent with those of previous studies of *A. niger* using the MPEC method (Driouch et al. 2010, 2012). However, during the second stage, we observed changes in the viscosity of the medium between days 13 and 21, which coincided with a peak in the production of the target compounds in the CZD and CZDM media modified with metallic mesh. These results are in agreement with previous ones pointing at the viscosity of the culture medium as the primary factor influencing the production of the target compounds (Johansen et al. 1998). Furthermore, polyester supports improved pigment production (Rengifo et al. 2023), demonstrating the feasibility and importance of interfacial interaction studies in fermentation, as described (Rouxhet 1991).

Significant improvements in the extract production were observed when various culture media and inert supports were used. Specifically, CZD and CZDM culture media demonstrated superior performance, yielding higher extract production than that reported in previous studies (Morales-Sánchez et al. 2021). Fermentation in PDB and Czapek-Dox culture media without any modification favored the production of **1** and **3**, respectively. The addition of talcum powder to different culture media yielded unexpected results regarding the production of the biocompounds of interest, possibly due to a consequence associated with oxygen and mass transfer limitations. The addition of an inert support (Metallic Mesh) led to the production of **2** and **4** in Czapek-Dox and the Modified Czapek-Dox, respectively.

The antifungal extract CZD/MM9 showed results comparable to those reported for the *Aspergillus* sp. SPH2 extract (Morales-Sánchez et al. 2021). However, the performance of the PDB/W5 extract was within the range previously reported. The extract from day 21 with the modified Czapek Dox and the addition of the metallic mesh (inert support) was promising against the tick, with an increase in performance of approximately 12% compared to the described SPH2 extracts (Morales-Sánchez et al. 2021). This means that owing to the stimulation by fungal nucleation and subsequent generation of a biofilm around the metallic mesh, there was an increase in the production of **4**.

## Conclusions

In conclusion, this study highlights the potential of improving the cultivation conditions of *Aspergillus* sp. SPH2, with implications extending beyond this specific fungus to other endophytic species. The methodological approach applied through experimentation with various latest-generation fermentation techniques introduced a valuable framework for the enhancement of the investigated technologies. Notably, modifications to the culture conditions for *Aspergillus* sp. SPH2 exerts profound effects on the production and biosynthesis of secondary metabolites, resulting in improved biocidal properties against *Botrytis cinerea* and *Hyalomma lusitanicum*. These findings highlight the versatility and promise of fungal biotechnology for both biocompound production and potential biocontrol applications, thereby offering sustainable solutions in agricultural and broader ecological contexts.

## Materials and methods

### Plant material

*Bethencourtia palmensis* were collected from Barranco del Rio, Abona (Tenerife, Spain) (28°34′10″ N, 16°18′48″ W). Within 48 h of collection, samples were placed in sterile polybags and transported in a box container under refrigeration until isolation. As indicated by Morales et al. (Morales-Sánchez et al. 2021), molecular characterization of the fungal strain *Aspergillus* sp. SPH2 was performed using a sequence search against the NCBI databases. The findings revealed that the strain exhibits similarities with the species within the Circumdati group, specifically *A. ochraceus* (GENBANK accession number KX901282.1) and *A. westerdijkiae* (GENBANK accession number KY608057.1).

### Cultivation of *Aspergillus* sp. SPH2 for extract preparation

*Aspergillus* sp. SPH2 was cultivated on PDA solid medium for eight days at 25 °C. Sterile water (10 mL) was added to each Petri dish to obtain a spore suspension for subsequent counting in a modified Neubauer chamber and then, 216 Erlenmeyer Flasks (100 mL) were prepared (72 primary each with their respective replicates), in which 72 flasks were prepared with 50 mL of Czapek-Dox liquid media ([CZD: NaNO_3_ (2 g/L), KH_2_PO_4_ (5 g/L), MgSO_4_ (0.5 g/L), FeSO_4_ (0.01 g/L), ZnSO_4_ (0. Three g/L), and glucose (30 g/L)], 72 with 50 mL of modified Czapek-Dox-Yeast liquid medium ([CZDM: NaNO_3_ (2 g/L), KH_2_PO_4_ (5 g/L), MgSO_4_ (0. 5 g/L), FeSO_4_ (0.01 g/L), ZnSO_4_ (0.003 g/L), yeast extract (1 g/L) and glucose (60 g/L)] and 72 others with 50 mL with Potato Dextrose Broth (Sigma-Aldrich). Inoculation with 1 × 10^6^ spores/mL was then performed. Three flasks were sampled on days 1, 2, 5, 9, 13, 16, 19, and 21 of the incubation. The culture medium was separated from the mycelium, dried in an oven at 40 °C for 24 h, and weighed.

### Modification of the fermentation conditions

Talcum powder (10 g/L) with a particle size between 44 and 35 µm (Fisher Chemical) was added to 72 of the 216 Erlenmeyer Flasks prepared (24 for each medium) according to the specifications of the optimal value evaluated and presented by Antecka et. al. (Antecka et al. 2016). Furthermore, a stainless steel (INOXIA) metal mesh 304 L with a pore size of 40 MESH and a surface of 0.044 m^2^ was added to the bottom of 72 Erlenmeyer’s following a design similar to that described by Francis et. al. (Francis et al. 2020).

### Extract preparation

The culture media was filtered through a paper filter using a Buchner funnel to separate the mycelium, submitted to exhaustive liquid/liquid extraction with ethyl acetate (3 × EtOAc), dried over Na_2_SO_4_, and concentrated under reduced pressure to obtain crude SPH2 extracts.

### Compound identification and quantification

Qualitative and quantitative determination of the ethyl acetate extract was performed by gas chromatography-mass spectrometry (GC–MS) using a Shimadzu GC-2010 gas chromatograph coupled to a Shimadzu GCMS-QP2010 Ultra mass detector (electron ionization, 70 eV). Sample injections (1 µL) were carried out using an AOC-20i instrument equipped with a 30 m × 0.25 mm i.e., capillary column (0.25 μm film thickness) Teknokroma TRB-5 (95%) Dimethyl- (5%) diphenylpolisiloxane. The working conditions were as follows: split ratio (20:1); injector temperature, 300 °C; temperature of the transfer line connected to the mass spectrometer, 250 °C; initial column temperature, 70 °C; and heating to 290 °C at 6 °C/min. Electron ionization mass spectra and retention data were used to assess the identity of the compounds by comparing them with those found in the Wiley 229 and NIST (version 17) mass spectral databases. All the extracts (4 µg/µL) were dissolved in 100% DCM for injection.

### Bioassays

#### Antifungal activity

*Botrytis cinerea* was obtained from the fungal collection at the Instituto de Productos Naturales and Agrobiologia-CSIC (Santa Cruz de Tenerife, Spain). Based on the study of Morales et. al. (Morales-Sánchez et al. 2021), the mycelial growth inhibition test was performed in 12-well Falcon plates using a modified agar-dilution method with 0.05 mg/mL methyltetrazolium salts (MTT). Extracts dissolved in ethanol (EtOH) were tested at various concentrations (extracts at 1, 0.5, 0.25, and 0.1 mg/mL) before being incorporated into the culture medium and poured into the plates. For each concentration tested, a series of test solutions was prepared in potato dextrose agar (PDA) and MTT, and 300 µL was added to each well. EtOH was used as a negative control and all treatments were replicated four times. After 48 h of incubation in the dark at 27 °C, fungal colonies were digitalized and measured using ImageJ (http://imagej.nih.gov/ij/). Percent inhibition (%I) was calculated as %I = (C T/C) 100, where C represents the diameter of the control colonies, and T represents the diameter of the test colonies (Parra Amin et al.^2021^). Data were analyzed using STATGRAPHICS statistical analysis software (Centurion XVIII), and EC_50_ values (effective dose to achieve 50% inhibition) were calculated using a regression curve of mycelial growth inhibition versus log dose.

### Ixodicidal activity

Female *Hyalomma lusitanicum* ticks were collected from their hosts (red deer) in central Spain (Finca La Garganta, Ciudad Real) and kept at 22–24 °C and 70% relative humidity until oviposition and egg hatching. The resulting larvae (4–6 weeks old) were used in this bioassay (Ruiz-Vásquez et al. 2017). The preparation process of the extract and its different concentrations entails adding it to 25 mg of cellulose, which is followed by the evaporation of the solvent utilized. Three replicates of 20 larvae were used for each test. Under the conditions described above, dead ticks were counted using a binocular magnifying glass 24 h after contact with treated cellulose. Larvicidal activity data were presented as percent mortality corrected using the Orelli-Schneider formula (Abou Lila et al. 2015). Probit Analysis was used to calculate effective lethal doses (LC_50_) (Sakuma 1998) using STATGRAPHICS Centurion XVI, version 16.1.02.

## Data Availability

Experimental data are available from the Biopesticides Group-CSIC database (contact: azu@ica.csic.es).
